# Thermogenic recruitment of brown and brite/beige adipose tissues is not obligatorily associated with macrophage accretion or attrition

**DOI:** 10.1152/ajpendo.00352.2020

**Published:** 2020-12-07

**Authors:** Nathalie Boulet, Ineke H.N Luijten, Barbara Cannon, Jan Nedergaard

**Affiliations:** Department of Molecular Biosciences, The Wenner-Gren Institute, Stockholm University, Stockholm, Sweden

**Keywords:** brown adipose tissue, cold, macrophages, obesity

## Abstract

Cold- and diet-induced recruitment of brown adipose tissue (BAT) and the browning of white adipose tissue (WAT) are dynamic processes, and the recruited state attained is a state of dynamic equilibrium, demanding continuous stimulation to be maintained. An involvement of macrophages, classical proinflammatory (M1) or alternatively activated anti-inflammatory (M2), is presently discussed as being an integral part of these processes. If these macrophages play a mediatory role in the recruitment process, such an involvement would have to be maintained in the recruited state. We have, therefore, investigated whether the recruited state of these tissues is associated with macrophage accretion or attrition. We found no correlation (positive or negative) between total UCP1 mRNA levels (as a measure of recruitment) and proinflammatory macrophages in any adipose depot. We found that in young chow-fed mice, cold-induced recruitment correlated with accretion of anti-inflammatory macrophages; however, such a correlation was not seen when cold-induced recruitment was studied in diet-induced obese mice. Furthermore, the anti-inflammatory macrophage accretion was mediated via β_1_/β_2_-adrenergic receptors; yet, in their absence, and thus in the absence of macrophage accretion, recruitment proceeded normally. We thus conclude that the classical recruited state in BAT and inguinal (brite/beige) WAT is not paralleled by macrophage accretion or attrition. Our results make mediatory roles for macrophages in the recruitment process less likely.

**NEW & NOTEWORTHY** A regulatory or mediatory role—positive or negative—for macrophages in the recruitment of brown adipose tissue is presently discussed. As the recruited state in the tissue is a dynamic process, maintenance of the recruited state would need persistent alterations in macrophage complement. Contrary to this expectation, we demonstrate here an absence of alterations in macrophage complement in thermogenically recruited brown—or brite/beige—adipose tissues. Macrophage regulation of thermogenic capacity is thus less likely.

## Graphical abstract

**Figure:**
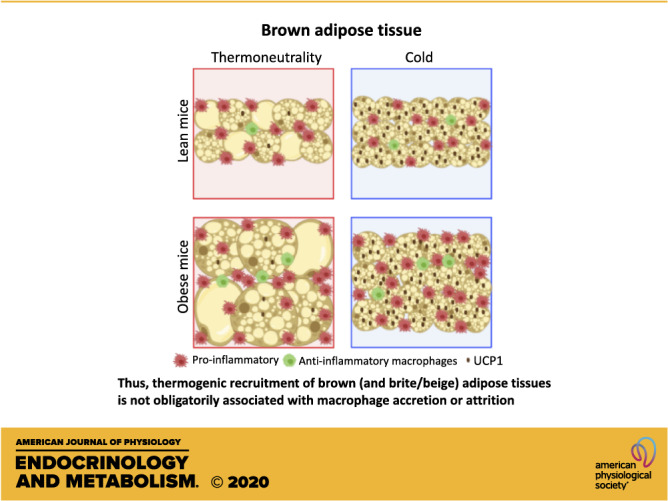

## INTRODUCTION

Maintenance of the recruited state of brown adipose tissue (BAT) is a dynamic equilibrium. Under natural conditions, the recruitment process is successive acclimation to decreasing environmental temperatures and not a sudden initiation of a transition phase. However, in an experimental cold-induced recruitment process, cell proliferation and differentiation take place abruptly over a period of some 3–4 wk to achieve a new level of thermogenic capacity. This process is generally accepted to be mediated by the sympathetic nervous system. The recruited state is, however, not inherently stable. Rather, the sympathetic nervous system must continue to constantly stimulate the tissue in order to maintain the increased number of cells and the higher degree of thermogenic differentiation. In the absence of this continuous stimulus, the tissue atrophies (1–7).

Although a direct effect of sympathetic stimulation on brown adipocyte progenitor proliferation and brown adipocyte differentiation is classically assumed, participation of paracrine mediators in the recruitment process is also discussed. Since the recruited state is dynamic, any such additional/alternative mediator of the recruitment process would be expected to remain constantly elevated during a maintained recruited state. The possibility that the immune system, particularly macrophages, is involved as a mediator or indeed is essential for, or otherwise controls, the recruitment process in brown—and perhaps particularly in brite/beige—adipose tissues has received much attention in recent years. Both negative and positive effects of the immune system (macrophages) on thermogenic recruitment have been suggested, as reviewed in detail (8).

A negative role of macrophages has been ascribed to the classical (M1, type 1) or proinflammatory macrophages. This negative effect has been suggested to occur particularly through their secretion of proinflammatory cytokines such as TNFα that can suppress UCP1 expression (9, 10), an effect reported to be enhanced by an increase in the number of these macrophages in BAT in obese mice (11). An influence of these macrophages on the cold-induced recruitment process should be evident as a decreased number of proinflammatory macrophages and thus a lower expression of the genes for the corresponding inhibitory cytokines. An increased UCP1 expression in the cold would thus follow from the reduction in inhibitory cytokines.

Alternatively, BAT recruitment and browning of white adipose tissue (WAT) have been suggested to be affected positively or even fully mediated by anti-inflammatory (M2, type 2, alternatively activated) macrophages. This could occur in different ways. These macrophages have been suggested to be activated in the tissue in the cold following release of norepinephrine from the sympathetic nervous system and interaction of norepinephrine with the macrophages. Secreted substances (cytokines, chemokines, and lipids) would then interact with adipocytes or preadipocytes to promote browning (12–18). The possibility that these macrophages directly stimulate BAT (or brite/beige adipose tissue) by release of macrophage-synthesized norepinephrine was proposed (19–22) but this suggestion would seem difficult to sustain (23). However, these macrophages could also influence sympathetic stimulation by taking up norepinephrine and possibly either degrading it or releasing it (24–26). Certain macrophages have also been suggested to be needed to maintain sympathetic innervation as such (27). In addition (or alternatively), anti-inflammatory macrophages have been suggested to release acetylcholine that would interact with cholinergic receptors on the brite/beige adipocytes themselves (28, 29) and in this way stimulate thermogenic recruitment.

However, the macrophage-related models for recruitment of thermogenic adipose tissues summarized earlier, together with the dynamic equilibrium nature of the recruited state, imply that the recruited state should be associated with marked alterations in the steady-state numbers of macrophages in the tissue: an attrition of proinflammatory and/or an accretion of anti-inflammatory macrophages. To investigate this hypothesis, we have examined here macrophage attrition and accretion in physiological states that have marked differences in the state of recruitment of BAT and brite/beige (inguinal) WAT (as well as epididymal WAT). When the results were analyzed from a physiological perspective, we failed to find evidence of an obligatory mediatory role of macrophage accretion or attrition in maintenance of BAT and brite/beige adipose tissue in a recruited state.

## MATERIALS AND METHODS

### Animals

All experiments were approved by the Animal Ethics Committee of the North Stockholm Region.

#### Chow-diet study.

Eleven weeks old C57BL/6J male mice were bought from Janvier (France). At 12 wk of age, the mice were single-caged. Some were kept at room temperature (21°C, referred to as semi-cold) or pre-cold-acclimated at 18°C for 1 wk. Then, half of the mice from 21°C was moved to 30°C (referred to as thermoneutral), half kept at 21°C, and all mice from 18°C were moved to cold (4°C) for 4 wk (referred to as cold-acclimated). The mice had free access to chow food (R70, Lactamin) and water, on a 12:12-h light-dark cycle.

#### β_1_/β_2_-Adrenergic receptor KO.

Twelve weeks old male β_1_/β_2_-adrenergic receptor knockout (β_1_/β_2_-AR KO) (30) mice (mixed background 129/SvJ, C57BL/6J, DBA/2, and FVB/N) and corresponding wild-type (i.e., not littermates but from a parallel line) were single-caged and housed at 21°C or pre-cold-acclimated at 18°C for 1 wk. Then, the mice were moved to 30°C or to 4°C for 4 wk. The mice had free access to chow food (R70, Lactamin) and water, on a 12:12-h light-dark cycle.

#### β_3_-Adrenergic receptor KO mice.

Briefly, 13- to 16-wk-old male β_3_-adrenergic receptor KO (β_3_-AR KO) (31) mice on the FVB/N background and corresponding wild-type (i.e., not littermates but from a parallel line) were single-caged and housed at 21°C or pre-cold acclimated at 18°C for 1 wk. Then, the mice were moved to 30°C or to 4°C for 4 wk. The mice had free access to chow food (R70, Lactamin) and water, on a 12:12-h light-dark cycle.

#### High-fat diet study.

Eleven weeks old C57BL/6J male mice were bought from Janvier (France). At 12 wk of age, the mice were single-caged and kept at 30°C on a 12:12-h light-dark cycle for 12 wk. The mice had free access to high-fat diet (45% calories from fat, Research Diets D12451) and water. According to the manufacturer, the fatty acid composition of the diet was 19% C16, 10% C18, 34% C18:1, 29% 18:2, 2% 18:3, and 6% other fatty acids. After 12 wk on high-fat diet, 2/3 of the mice were kept at 30°C and 1/3 was moved to 18°C for 1 wk pre-cold-acclimation. The mice from 18°C were then transferred to 4°C, whereas half of the mice from 30°C were moved to 21°C and half were kept at 30°C for 4 wk. All mice were fed the 45% high-fat diet.

#### Final treatment of all mice.

One day before the end of the experiments, body composition was measured (EchoMRI-100, Echo Medical Systems). The day after, the mice were euthanized with CO_2_ followed by cervical dislocation. Immediately after death, the mice were perfused with 20 mL of PBS through the left ventricle to remove blood from tissues. Interscapular BAT (BAT), inguinal WAT (ingWAT; lymph nodes were removed), and epididymal WAT (epiWAT) were dissected quantitively and weighed; a small piece of each tissue was immediately snap-frozen in liquid nitrogen and the main part was again weighed and kept in DMEM high glucose (4,500 mg/L, Sigma-Aldrich) for immediate stromal-vascular fraction isolation.

### Adipose Tissue Stromal-Vascular Fraction Cell Isolation

The BAT, ingWAT, and epiWAT samples were minced with scissors and digested in a buffer (100 mM HEPES, 123 mM NaCl, 5 mM KCl, 1.3 mM CaCl_2_, 5 mM glucose, 1.5% BSA, ddH_2_O, pH 7.4) containing 1 mg/mL collagenase type II (Sigma-Aldrich) for 30–45 min in a shaking water bath at 37°C. After the digestion, the suspension was filtered through a 250-μm nylon filter and kept on ice for 15 min. Floating mature adipocytes were discarded, and the remaining cell suspension was filtered through a 37-μm nylon filter and centrifuged at 1,300 rpm for 10 min at room temperature. The cell pellet was suspended in 37°C DMEM high-glucose (4,500 mg/L, Sigma-Aldrich) and centrifuged at 1,300 rpm for 10 min at room temperature. The cell pellet was suspended in 37°C erythrocyte lysis buffer (0.15 M NH_4_Cl, 10 mM NaHCO_3_, 0.1 mM EDTA, ddH_2_0, pH 7.3) and centrifuged at 1,300 rpm for 10 min at 4°C. This final pellet, the stromal-vascular cell (SVC) fraction, was suspended in cold FACS buffer (PBS with 0.5% BSA and 2 mM EDTA) and counted in a Bürker chamber using Trypan blue exclusion. The total number of cells per adipose depot was calculated from the weight of the digested tissue as a fraction of the total tissue weight.

### Flow Cytometry Analyses

The SVCs were preincubated with mouse BD FcR block (clone 2.4G2, BD Biosciences) for 5 min at 4°C and then incubated with fluorescent-labeled monoclonal anti-mouse antibodies (F4/80 BV421 clone BM8, CD206 FITC clone C068C2, CD301 PE clone LOM-14 from BioLegend; CD86 APC clone B7-2 from BD Biosciences), or respective immunoglobulin controls (immunoglobulins and antibodies dilution 1:100) in the FACS buffer for 30 min at 4°C in the dark. The cells were washed by adding PBS and centrifuged at 1,300 rpm for 10 min at 4°C); the labeled cells were fixed using BD CellFIX (BD Biosciences) and analyzed by flow cytometry using a FACS Verse flow cytometer (BD Biosciences) and FlowJo software.

### RNA Isolation, cDNA Synthesis, and qPCR

The frozen tissues were homogenized in Tri Reagent (Sigma-Aldrich). RNA was extracted using the chloroform-isopropanol method according to the manufacturer’s instructions. RNA concentration was measured using NanoDrop 1000 Spectrophotometer (Thermo Scientific). RNA (500 ng) was reverse-transcribed using the High Capacity cDNA kit (Life Technologies) in a total volume of 20 μL, then diluted 10 times in water. Gene-specific primers (Sigma-Aldrich) were premixed with 11 μL of SYBR Green JumpStart Taq Ready Mix (Sigma-Aldrich) to a final concentration of 0.3 μM; primer sequences are listed in Table 1. The amplification reaction was done in duplicate on 2 μL of the diluted cDNA in a final volume of 13 μL in 96-well reaction plates (Bio-Rad) on a Bio-Rad CFX Connect Real-Time system. Thermal cycling conditions were 2 min at 50°C, 10 min at 95°C, and 40 cycles of 15 s at 95°C and 1 min at 60°C. Values were normalized to the levels of TFIIB (for BAT), 18S (for ingWAT), and Hprt (for epiWAT) mRNA. Different normalization genes were used for the different tissues to ensure stable normalization levels between the different physiological conditions, as shown in the figures. The Δ*C_T_* method (2^−Δ^*^CT^*) was used to calculate relative changes in mRNA abundance. Values were then normalized and presented as fold-change over the 30°C group.

**Table 1.** Primer sequences

Gene	Forward	Reverse
Arg1	CTGAGCTTTGATGTCGACGG	TCCTCTGCTGTCTTCCCAAG
Hprt	CTGGTTAAGCAGTACAGCCCCAA	CGAGAGGTCCTTTTCACCAGC
Il1β	TGTAATGAAAGACGGCACACC	TCTTCTTTGGGTATTGCTTGG
Il10	TTGAATTCCCTGGGTGAGAAG	TCCACTGCCTTGCTCTTATTT
Il6	CATTTGTGGTTGGGTCAGG	AGTGAGGAACAAGCCAGAGC
iNos	TCTCCCTTTCCTCCCTTCTT	CTTCAGTCAGGAGGTTGAGTTT
Mgl1	TGAGAAAGGCTTTAAGAACTGGG	GACCACCTGTAGTGATGTGGG
Mrc1	CAAACTGGGGGAAAGGCTAT	TTGCCACCAATCACAACTACA
TFIIB	TGGAGATTTGTCCACCATGA	GAATTGCCAAACTCATCAAAACT
Tnfα	CCAAGGCGCCACATCTCCCT	GCTTTCTGTGCTCATGGTGT
Ucp1	GGCCTCTACGACTCAGTCCA	TAAGCCGGCTGAGATCTTGT
18S	AGTCCCTGCCCTTTGTACACA	CGATCCGAGGGCCTCACTA

### Statistical Analyses

Values are given as means ± SE for (*n*) independent experiments (mice). Statistical analyses were performed using Prism (GraphPad software). Comparisons between groups were analyzed by One-way ANOVA followed by Tukey’s or Sidak’s post test or two-way ANOVA followed by Sidak’s post test. Correlations were analyzed by Spearman test. Differences were considered statistically significant when *P* < 0.05.

## RESULTS

Since macrophages have been suggested to be actively—positively or negatively—involved in the recruitment of BAT and brite/beige adipose tissue and since this recruited state is a dynamic equilibrium requiring persistent stimulation to be maintained, we have investigated whether the physiologically induced dynamically recruited state is associated in a systematic way with alterations in macrophage numbers or activation state (anti- or proinflammatory). We have analyzed recruitment under two physiological paradigms: standard cold acclimation and in the interplay between diet-induced obesity and recruitment induced by cold acclimation of already obese mice.

### Are Macrophages Recruited to Adipose Tissues by Cold Acclimation?

**Figure 1.**
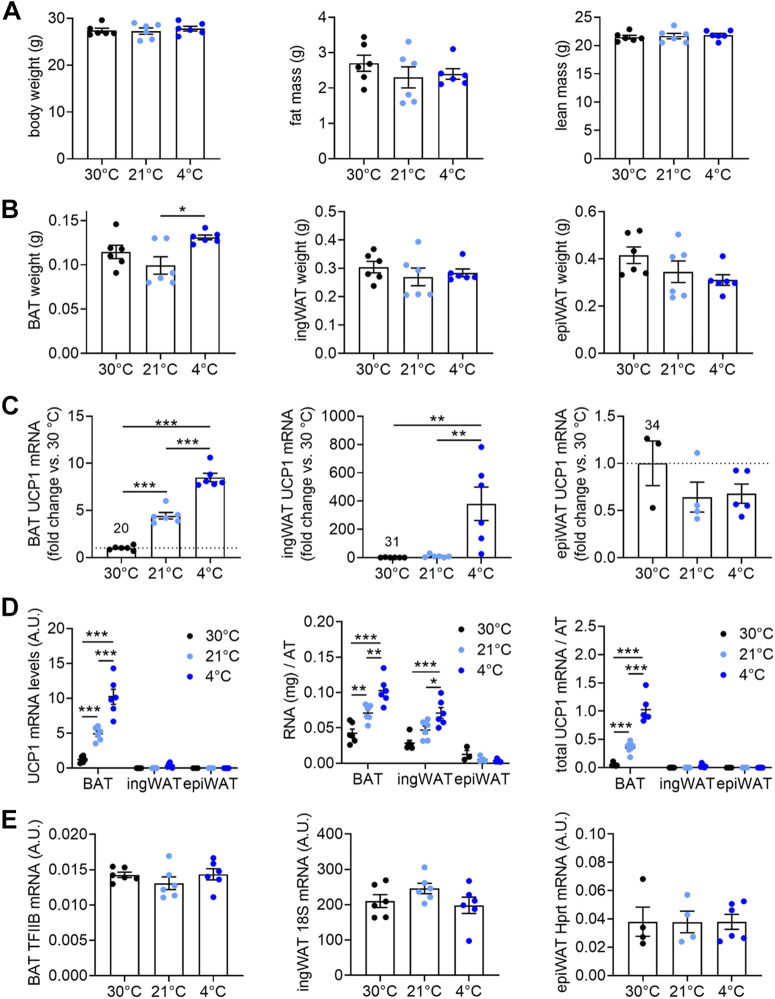
Effect of housing temperature on adipose tissue recruitment. Male C57BL/6J mice were fed a chow diet and acclimated to different temperatures (30°C, 21°C, and 4°C) for 4 wk, as detailed in methods. Final values are presented here. *A*: body weight, fat mass, and lean mass. *B*: wet weights of interscapular brown adipose tissue (BAT), inguinal white adipose tissue (ingWAT), and epididymal white adipose tissue (epiWAT). *C*: UCP1 gene expression determined by quantitative polymerase chain reaction (qPCR) in BAT, ingWAT, and epiWAT, expressed as fold change over the 30°C group. The values given above the 30°C bars indicate the mean *Ct* value for that condition in each tissue. *D*: UCP1 mRNA levels in BAT, ingWAT, and epiWAT; values are 2^−^*^Ct^* ×1,000,000 (*left*). Total amount of RNA in BAT, ingWAT, and epiWAT depots (*middle*). Total amounts of UCP1 mRNA in BAT, ingWAT, and epiWAT (*right*); data were obtained by multiplying the data in the *left* with the data in the *middle*. *E*: reference gene expression levels in BAT (TFIIB), ingWAT (18S), and epiWAT (Hprt) related to data in Figs. 1*C* and 2; values are antilog-transformed *Ct* values (2^−^*^Ct^* × 1,000,000). Statistics for all panels: *n* = 3–6 mice/group, means ± SE, one-way ANOVA and Tukey’s (Sidak’s for *D*) multiple comparisons test, *P < 0.05, **P < 0.01, ***P < 0.001.

We investigated initially the potential role of macrophages in the dynamic maintenance of recruited BAT, as well as in browning maintenance in WAT. Particularly, we wanted to examine whether the recruited state was associated with a systematic accretion or attrition of the number of pro- and anti-inflammatory macrophages in the adipose tissue depots. Mice were therefore acclimated to different temperatures: thermoneutrality (30°C), semi-cold (animal house standard room temperature of 21°C), and cold (4°C). The different ambient temperatures did not affect body weight, total fat, and total lean mass (Fig. 1*A*), and there were no marked effects of ambient temperature on the wet weight of the three adipose depots investigated (Fig. 1*B*): the classical BAT, the inguinal adipose depot (ingWAT)—that has the ability to “brown,” i.e., to express the UCP1 gene (i.e., brite/beige tissue)—and the epididymal WAT depot (epiWAT), a white adipose depot that is practically unable to physiologically express the UCP1 gene (see also next section).

#### BAT is the main site of cold-induced recruitment of UCP1.

As expected, the three different housing temperatures were associated with different UCP1 gene expression levels in BAT and ingWAT but not in epiWAT (Fig. 1, *C* and *D*); the expression levels of the reference used for each tissue are shown in Fig. 1*E* and were not affected by housing temperature.

It is seen (Fig. 1*C*) that the increased recruitment drive at 21°C and 4°C was associated with an increase in UCP1 mRNA levels in BAT, up to an eightfold increase. The relative increase due to cold acclimation was very much larger (around 400-fold) in ingWAT than in BAT. In epiWAT, no increase in UCP1 mRNA levels was seen. However, these values represent relative increases; quite a different impression is obtained when the absolute values for UCP1 mRNA levels are compared. These levels (calculated as 2^−^*^Ct^* × 1,000,000, i.e., principally per mg RNA) were much higher in BAT than in ingWAT (900-fold at 30°C and ∼25-fold higher at 4°C) and very much higher in BAT than in epiWAT (∼18,000-fold at 30°C increasing to 150,000-fold at 4°C) (Fig. 1*D*, *left*).

However, not only does the gene expression level of UCP1 increase in the cold, the total amount of RNA also increases in BAT (as has long been realized (32)) and also in ingWAT, but not in epiWAT (Fig. 1*D*, *middle*). Thus, the total amount of UCP1 mRNA in the three tissues in the three conditions can be calculated (Fig. 1*D*, *right*). Although UCP1 mRNA does not in itself produce heat (33), the total amount of UCP1 mRNA in a given adipose depot is a rather good predictor under steady-state conditions of total UCP1 protein amounts and thus of thermogenic capacity (44). According to this, the thermogenic capacity of BAT increased sevenfold in semi-cold and 20-fold in standard cold, as compared with thermoneutral conditions, and that of the total inguinal WAT 20-fold and 900-fold, respectively. Nonetheless, the total amount of UCP1 mRNA in total interscapular BAT in these C57BL/6J mice was 1,600 times higher than that in total ingWAT at 30°C, 550 times higher at 21°C, and 35 times higher at 4°C. Thus, based on total UCP1 mRNA amounts as proxies for total thermogenic capacities, the contribution of the brite/beige tissues to cold-acclimation-recruited heat production would be very modest in these mice.

#### Apparent repression by cold acclimation of inflammatory markers in BAT.

**Figure 2.**
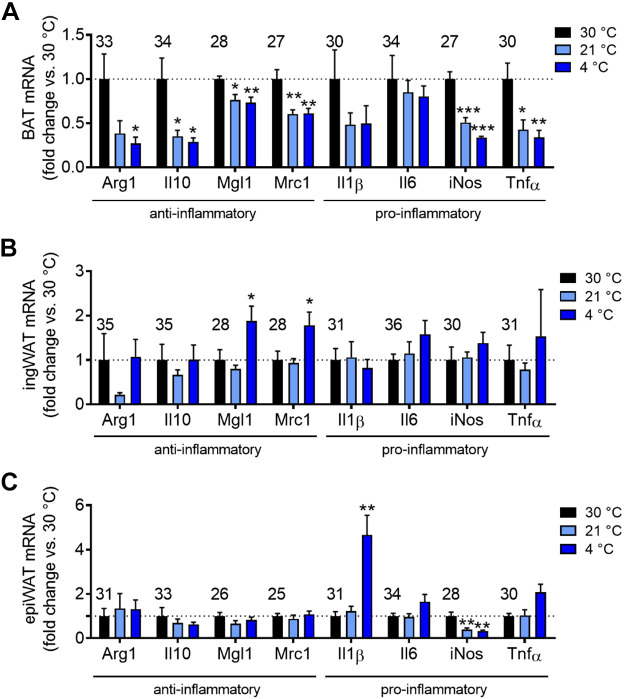
Apparent effect of cold acclimation on inflammatory status of adipose tissues. The adipose tissues of the mice characterized in Fig. 1 were analyzed for pro- and anti-inflammatory markers. Gene expression of anti-inflammatory markers (Arg1, Il10, Mgl1, Mrc1) and proinflammatory markers (Il1β, Il6, iNos, Tnfα); brown adipose tissue (BAT) (*A*), inguinal white adipose tissue (ingWAT) (*B*), and epididymal white adipose tissue (epiWAT) (*C*). The values were calculated versus the reference genes shown in Fig. 1*E*. Results are expressed as fold change over the 30°C group. The value above each gene is the mean *Ct* value for the gene at 30°C; higher values thus indicate lower expression levels. Statistics for all genes: *n* = 4–6 mice/group, means ± SE, one-way ANOVA and Sidak’s multiple comparisons test, *P < 0.05, **P < 0.01, ***P < 0.001 compared with 30°C.

To characterize the association between macrophages and the thermogenic state of the tissues, we determined the expression of the genes of several generally accepted anti-inflammatory (Arg1, Il10, Mgl1, and Mrc1) and proinflammatory (Il1β, Il6, iNos, and Tnfα) macrophage markers in whole tissue (this may thus also include parenchymal cell gene expression). We are well aware that such a division is somewhat blunt. In general, the expression levels of all examined pro- or anti-inflammatory genes were very low on a tissue basis, in that the *Ct* values were generally ≥30 (Fig. 2).

For comparisons, the results were normalized to the thermoneutral (30°C) group. In BAT, most of the markers measured were decreased (statistically or with a clear visual tendency) upon semi-cold or cold acclimation, compared with the 30°C group, when expressed in this way, i.e., principally per mg RNA. This was thus the case for both the anti- (Arg1, Il10, Mgl1, and Mrc1), and the proinflammatory (iNos and Tnfα) markers (Fig. 2*A*). The markers were not further decreased by an increased degree of cold exposure (4°C vs. 21°C) (Fig. 2*A*). This is principally in agreement with the perception that most of the differentiation-inducing effects of cold on gene expression in BAT are achieved already under the semi-cold conditions encountered under “standard” animal house conditions; more intense cold acclimation results rather in tissue growth (44).

Although the association between increased tissue recruitment (Fig. 1) and a general repression of pro- and anti-inflammatory marker gene expression (Fig. 2*A*) is only correlative, the very consistent negative effect of cold acclimation on the macrophage-associated markers observed here would principally be in accordance both with the concept that macrophages are inhibitory for BAT recruitment—and with the possibility that macrophage depletion could contribute to BAT recruitment (see introduction).

In contrast to the consistent results in BAT, we did not observe a systematic change in macrophage-associated gene expression in ingWAT. There was a modest increase in the expression of the anti-inflammatory markers Mgl1 and Mrc1 upon cold acclimation (Fig. 2*B*). This general lack of effect was unexpected, considering the possibility that alternatively activated (anti-inflammatory) macrophages could be mediatory for the browning process (8) and would thus be expected to remain high in recruited ingWAT, to mediate or maintain the recruited state.

Similarly, in epiWAT, there was no consistent effect of cold acclimation on gene expression levels. Il1β and iNos, two proinflammatory markers, were increased and decreased, respectively, in the cold (Fig. 2*C*).

Thus, based on relative levels of gene expression, BAT appears conspicuous in showing consistent negative effects of cold-induced recruitment on the expression of both pro- and anti-inflammatory markers to a similar extent. This would be in apparent agreement with a negative regulatory role of macrophages in BAT recruitment.

#### Effect of cold acclimation on macrophage accumulation.

The above data on macrophage-associated gene expression would imply that BAT recruitment was associated with a marked lowering of the number of macrophages in the tissue. However, the expression of certain pro- and anti-inflammatory markers in the tissues may include expression in macrophages as well as in other cells, including the parenchymal cells (the adipocytes, e.g., see Ref. 34). To address the direct relationship between macrophage accumulation and cold acclimation-induced recruitment in the adipose tissues, we proceeded to identify and count the macrophages, both pro- and anti-inflammatory.

**Figure 3.**
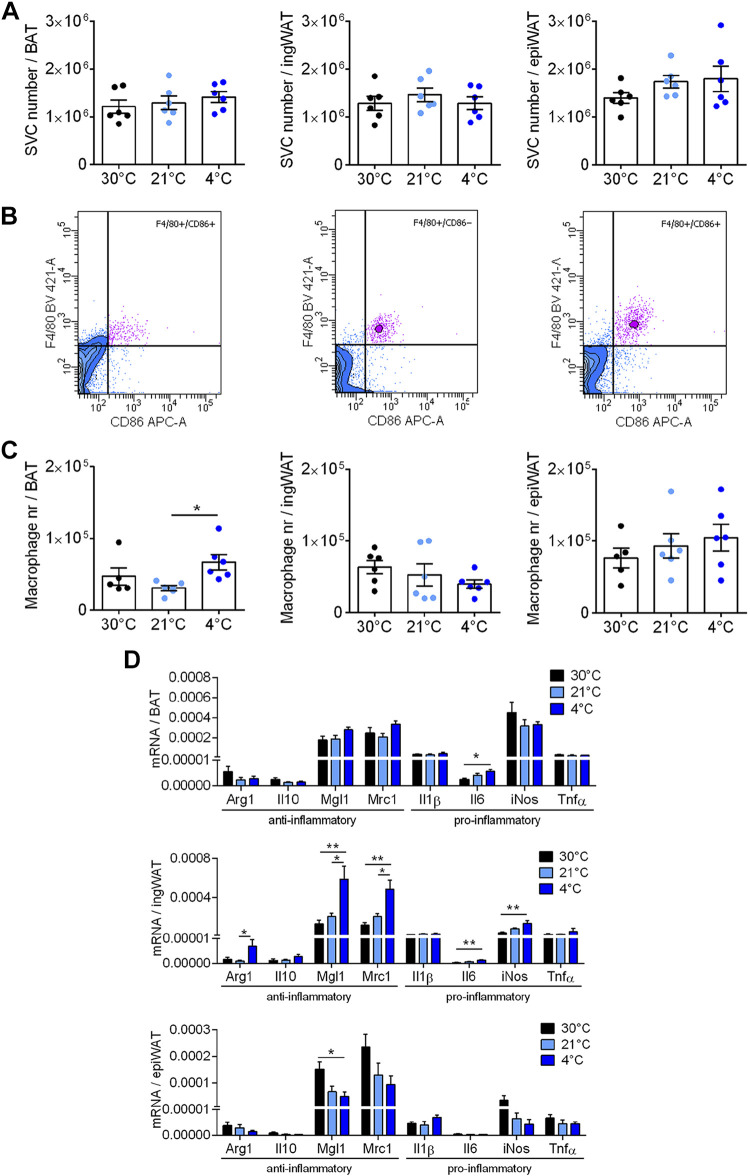
No macrophage attrition during cold-induced recruitment. The stromal-vascular fraction was isolated from the adipose tissue depots characterized in Fig. 1. *A*: recovered number of stromal-vascular cells (SVC) per brown adipose tissue (BAT), inguinal white adipose tissue (ingWAT), and epididymal white adipose tissue (epiWAT) depot. *B*: representative flow cytometry contour plots of F4/80 and CD86; all three plots are from one mouse acclimated to 30°C. Macrophages were defined as F4/80+ and CD86+ cells, with the borders set as illustrated here; the same borders were used for all depots analyzed. In the contour plots, violet dots indicate F4/80+/CD86+ cells, blue dots indicate non-F4/80+/CD86+. *C*: the effect of cold acclimation on the total number of macrophages recovered from the three tissues. *D*: Total levels of mRNA for the genes examined in Fig. 2 in BAT (*top*), ingWAT (*middle*), and epiWAT (*bottom*, one outlier was removed). The data were obtained in a similar way to that for UCP1 in Fig. 1*E* (*right*), i.e., by multiplying the levels of each mRNA by the total amount of RNA in the tissue (Fig. 1*E*, *middle*). Statistics for all panels: *n* = 3–6 mice/group, means ± SE, one-way ANOVA and Tukey’s (Sidak’s for *D*) multiple comparisons test, *P < 0.05, **P < 0.01.

For this, we isolated the stromal-vascular cells (SVCs) and counted the total number of recovered cells in the three depots. The total number of SVCs in each depot was rather similar in all three depots (about one million per depot), and was unaffected by housing temperature (Fig. 3*A*).

The stromal-vascular fraction consists of many different cell types (preadipocytes, endothelial cells, other immune cells, etc.) in addition to macrophages. Therefore, to characterize the macrophages in the tissues, flow cytometric analyses were performed on the fractions.

An example of the flow cytometric analysis is shown in Fig. 3*B*, with data from all three depots from one mouse acclimated at 30°C. Macrophages were defined as cells that were F4/80+ and CD86+; as seen, most of the F4/80+ cells were also CD86+. In Fig. 3*C*, the mean total number of macrophages recovered from the three tissues under the different temperature acclimation conditions is shown. The total number was rather similar in all three depots: ∼50,000, i.e., ∼5% of the total number of SVC. This low number of macrophages could partly explain the relatively low expression levels (high *Ct* values) seen in Fig. 2, this probably mainly reflects a dilution of macrophage mRNA levels by RNA from other cell types.

Notably, though, there was essentially no effect of the different environmental temperatures on the total number of macrophages, measured in this way, in any of the adipose depots. This was initially unexpected. Considering the clear effects of cold acclimation on the expression levels of analyzed genes, particularly in BAT (Fig. 2), a marked decrease in macrophage number would have been anticipated, or a large divergence between the gene expression data and the FACS data would need to be suggested.

However, the gene expression data in Fig. 2 were expressed as is usual, i.e., principally per mg RNA (even though all data were double-normalized to reference genes and thermoneutrality values). As shown earlier, the total amount of RNA is increased due to cold acclimation (Fig. 1*D*, *middle*). This increase is presumably due to the enhanced expression of the genes related to thermogenesis and also to a general augmentation of tissue structure: more ribosomes, etc. This would necessarily dilute any other mRNA. Thus, from a physiological point of view, it is the total mRNA amount of each gene in the tissues that is of interest, just as it is the total number of macrophages in the tissue that are counted in Fig. 3*C*.

Therefore, we calculated the total tissue expression of the pro- and anti-inflammatory genes in BAT, similarly to the calculation of total UCP1 gene expression in Fig. 1*D*. As seen (Fig. 3*D*), given this integrative approach, the result becomes qualitatively different: the apparent decrease in gene expression caused by cold acclimation was only apparent, and was the result of dilution by the extra RNA synthesized during tissue recruitment. When total mRNA levels were calculated, there was practically no effect of recruitment state, only a minor increase in Il-6. There is thus good agreement between the observation that the total number of macrophages found in the BAT was not markedly changed due to cold acclimation (Fig. 3*C*, *left*) and the observation that the total expression of pro- and anti-inflammatory genes was not markedly changed under these conditions (Fig. 3*D*).

In the ingWAT, when analyzed in this way, the predominant effect of cold acclimation was an enhanced expression of two anti-inflammatory markers, Mgl1 and Mrc1, as well as of two proinflammatory markers iNos and Il6. In the epiWAT, no enhancement of gene expression was observed, only a modest decrease in Mgl1 was indicated.

#### Accumulation of pro- or anti-inflammatory macrophages.

As there was an indication that the expression of anti-inflammatory genes was enhanced in the ingWAT during cold acclimation (Fig. 3*D*), we further examined the macrophage population. The macrophages within the adipose tissues can be considered to consist of both pro- and anti-inflammatory macrophages. We have followed the convention that the presence of CD301 and CD206 markers on these cells would define them as anti-inflammatory macrophages, while the absence of these markers would indicate proinflammatory macrophages, and we have thus analyzed the macrophage populations as being pro- or anti-inflammatory according to this.

**Figure 4.**
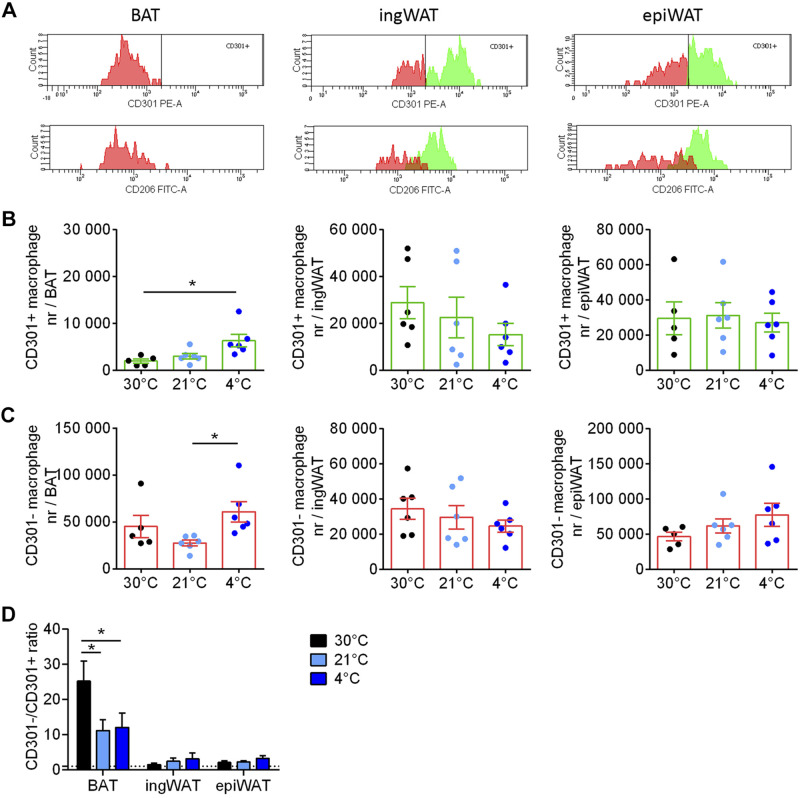
Effect of cold acclimation on accumulation of anti- and proinflammatory macrophages in adipose tissues. *A*: the cells that in Fig. 3*C* were identified as macrophages based on their levels of F4/80 and CD86 (the F4/80+/CD86+ cells) were further analyzed for markers of anti-inflammatory or proinflammatory macrophages, as illustrated here for one mouse acclimated to 30°C (same mouse as analyzed in Fig. 3*B*). For each adipose tissue, the top histogram shows the distribution of the fluorescence of CD301; cells above the gate are considered anti-inflammatory and are coloured green; those below the gate are colored orange and considered proinflammatory. The bottom histogram shows the fluorescence of these cells for CD206; thus, the CD301+ cells are still depicted here as green and the CD301− cells as orange. It is evident that the cells identified as anti-inflammatory based on the CD301 levels (green cells) largely distribute similarly (to the right) when analyzed for CD206, and those identified as proinflammatory based on the CD301 levels (orange cells) distribute to the left also for CD206. Cells are therefore largely either CD301+/CD206+ and considered anti-inflammatory or CD301−/CD206− and considered proinflammatory. The further analysis therefore utilizes only CD301 as a marker for pro- versus anti-inflammatory cells. Note that in the sample from brown adipose tissue (BAT) shown here, there were practically no anti-inflammatory cells. Number of F4/80+/CD86+/CD301+ (anti-inflammatory macrophages) (*B*) and number of F4/80+/CD86+/CD301− (proinflammatory macrophages) (*C*) per BAT (*left*), inguinal white adipose tissue (ingWAT, *middle*), and epididymal white adipose tissue (epiWAT, *right*). *D*: CD301−/CD301+ macrophages (pro-/anti-inflammatory) ratio in BAT, ingWAT, and epiWAT. Statistics for all panels: *n* = 6 mice/group; means ± SE, one-way ANOVA and Tukey’s (Sidak’s for *D*) multiple comparisons test, *P < 0.05.

In Fig. 4*A* (*top* row), we have labeled those cells that were classified as CD301− in orange and those that were CD301+ as green. When the same cells were analyzed for being CD206− or +, we found that practically all macrophages that were proinflammatory according to the CD301 definition (F4/80+/CD86+/CD301− macrophages), i.e., the orange cells in the top row, were also proinflammatory according to the CD206 definition (bottom row). Similarly, the cells that were CD301+ (green cells, top row) were generally also CD206+ (green cells, bottom row). Thus, in the following, only the CD301+ anti-inflammatory macrophage quantification is shown.

Remarkably, in BAT, only ∼5%–10% of the macrophages were F4/80+/CD86+/CD301+ and thus considered to be anti-inflammatory macrophages, whereas the F4/80+/CD86+/CD301− proinflammatory macrophages represented more than 90% of the tissue macrophage population. There was a gradual increase in the total number of anti-inflammatory macrophages in BAT as a result of cold acclimation, whereas the number of proinflammatory macrophages was essentially unchanged by cold (Fig. 4, *B* and *C*).

In ingWAT and epiWAT, the number of anti- (F4/80+/CD86+/CD301+) and proinflammatory (F4/80+/CD86+/CD301−) macrophages were more similar and were unaffected by housing temperature (Fig. 4, *B* and *C*).

It is evident from these numbers that there were distinct differences between the tissues regarding the pro-/anti-inflammatory macrophage ratio. In BAT, this ratio was high but was somewhat reduced during cold acclimation; in ingWAT and epiWAT, the ratio was close to 1 and not affected by cold acclimation (Fig. 4*D*). This remarkable difference between the macrophage populations in BAT versus the other adipose tissues is in reasonable agreement with the different levels of expression of what are considered pro- and anti-inflammatory genes (Fig. 3*D*). The proinflammatory marker iNos is expressed at a higher level in BAT than in ing/epiWAT, whereas at 4°C the anti-inflammatory markers Mgl1 and Mrc1 are expressed at somewhat higher levels in ingWAT than in BAT. The physiological significance of these differences in gene expression is not presently established.

### Significance of β-Adrenergic Receptors for Macrophage Population Alterations

Although the total macrophage number was not much changed, chronic cold exposure did increase the (low) number of CD301+ (anti-inflammatory) macrophages in BAT (Fig. 4*B*). Since cold-induced recruitment of the tissue is governed by the sympathetic nervous system, the possibility existed that the sympathetic nervous system also induced this accretion of anti-inflammatory macrophages and that it could be this accretion of macrophages that mediates the effect of cold acclimation on the tissue, as has been suggested (see introduction). This would be in accordance with reports concerning other tissues that the sympathetic nervous system and catecholamines could directly modulate macrophage phenotype toward an anti-inflammatory phenotype, through the β_2_-adrenergic receptor (13, 18).

Thus, to determine if the effects of housing temperature on macrophage populations were mediated by sympathetic nervous activity, the effect of cold acclimation on macrophage number was analyzed as in Fig. 4 in β-adrenoceptor knockout mice.

**Figure 5.**
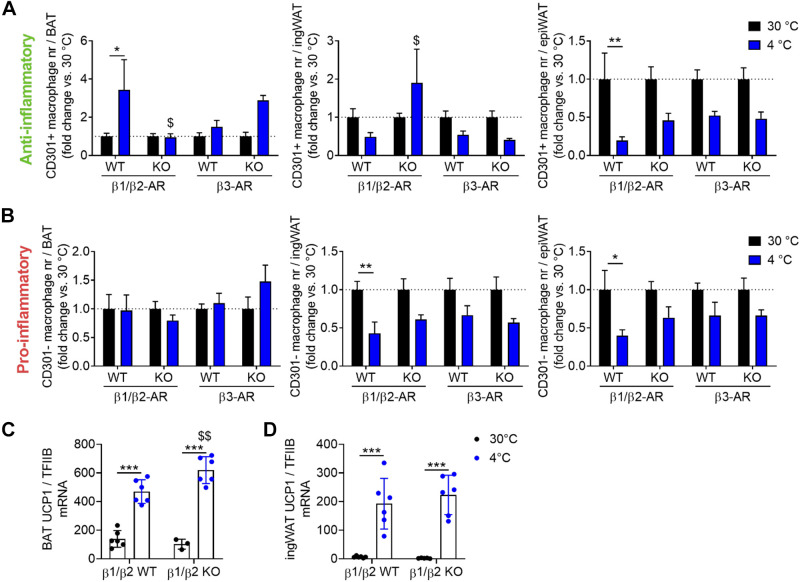
Cold-induced increase in anti-inflammatory macrophages in brown adipose tissue (BAT) is dependent on β_1_/β_2_-adrenergic receptors. Mice lacking β_1_/β_2_- or β_3_-adrenergic receptors and corresponding wild-types were exposed to 30°C or 4°C for 4 wk and the number of pro- or anti-inflammatory macrophages determined as detailed in methods and in preceding figures. *A*: relative anti-inflammatory macrophage number in brown adipose tissue (BAT, *left*), inguinal white adipose tissue (ingWAT, *middle*), and epididymal white adipose tissue (epiWAT, *right*). *B*: similar for proinflammatory macrophages. For each strain, the results were normalized to the average number in the thermoneutrality group. *C*: UCP1 mRNA levels in BAT for the β_1_/β_2_-knockout (KO) and corresponding wild-type mice examined in (*A*, *B: left*). *D*: UCP1 mRNA levels in ingWAT for the same mice as those examined in (*A*, *B*: *middle*). Statistics for all panels: *n* = 4–6 mice/group, means ± SE, two-way ANOVA and Sidak’s multiple comparisons test, *P < 0.05, **P < 0.01, and ***P < 0.001 for effect of cold acclimation; $, $$ similar for difference between KO and corresponding wild-type.

In β_1_/β_2_-adrenoceptor wild-type mice, that are on a very mixed genetic background, cold acclimation increased the number of CD301+ anti-inflammatory macrophages in BAT but did not have any effect on CD301− proinflammatory macrophages (Fig. 5, *A* and *B*, *left*) (as in the C57BL/6J mice described in Fig. 4*B*). In the β_1_/β_2_-adrenoceptor knockout mice, the cold-induced increase in anti-inflammatory macrophage number was abolished. In contrast, loss of the β_3_-adrenergic receptor was not associated with a loss of the ability of cold to increase anti-inflammatory macrophages. Thus, the β_3_-adrenoceptor was dispensable for the accretion of anti-inflammatory macrophages in BAT in the cold, but the β_1_/β_2_-adrenoceptors were essential.

In the ingWAT and epiWAT tissues [where cold acclimation did not markedly affect the pro- or anti-inflammatory macrophage number (Fig. 4, *B* and *C*)], the absence of the β_1_/β_2_- or of the β_3_-adrenoceptors did not systematically affect the response to cold acclimation, as compared with the respective wild-type (Fig. 5, *A* and *B*, *middle* and *right*).

These results thus demonstrate that the β_1_ and/or β_2_-adrenergic receptors are necessary for the increase of anti-inflammatory macrophage number in classical BAT. As the lack of β-adrenoceptors is global, these experiments cannot distinguish between the possibilities that the stimulatory adrenergic effect is directly on the macrophages or indirectly via other cells—but based on earlier experiments (13, 18), it is likely that this was a direct effect on the macrophages themselves.

Given the tenet that recruitment of anti-inflammatory macrophages should be an essential or determinative step in the recruitment of BAT thermogenic activity (see introduction), we examined whether the absence of an increase in anti-inflammatory macrophages affected thermogenic recruitment, evaluated as the cold acclimation-induced increase in UCP1 mRNA. As seen in Fig. 5*C*, this was not the case; nor did the absence of β_1_/β_2_-receptors affect UCP1 gene expression in the ingWAT (Fig. 5*D*) [where no cold-induced increase in anti-inflammatory macrophages had been evident (Fig. 5*A*)]. Thus, whereas β-adrenergic stimulation of anti-inflammatory macrophage accretion was observed here, we found no evidence that this is necessary for the BAT recruitment.

### Even in Diet-Induced Obesity, BAT Recruitment is Not Associated with Macrophage Accretion or Attrition

The mice examined in Figs. 1-4 for cold-induced effects on adipose tissue macrophage numbers were standard mice in that they were young and fed a standard chow diet. However, macrophage accumulation not only in WAT but also in BAT has been reported to be associated with diet-induced obesity (11, 35). To determine whether, under conditions of anticipated higher macrophage infiltration, the effects of cold-induced recruitment would include significant and perhaps mediatory alterations in macrophage complement, we first induced obesity in mice by long-term high-fat diet feeding (12 wk) at thermoneutrality. We then initiated the classical recruitment process in BAT (and ingWAT) by exposing the mice to semi-cold (21°C) and cold (4°C) conditions, for 4 wk as in Figs. 1-4, during which the mice were maintained on the high-fat diet.

Compared with the nonobese mice examined in Figs. 1-4, the diet-induced obese mice were necessarily older due to the feeding period. Thus, although they had been exposed to the recruiting temperature conditions for the same time period as the nonobese mice, their final age was 28 wk, as compared with 16 wk for the nonobese mice. Thus, direct comparisons between the groups may possibly be confounded by an effect of age. However, since adult mice on chow diet are rather stable with respect to physiological characteristics, we consider that most differences between the two groups are due to obesity rather than to age, and some comparisons are therefore made between the nonobese and obese mice.

**Figure 6.**
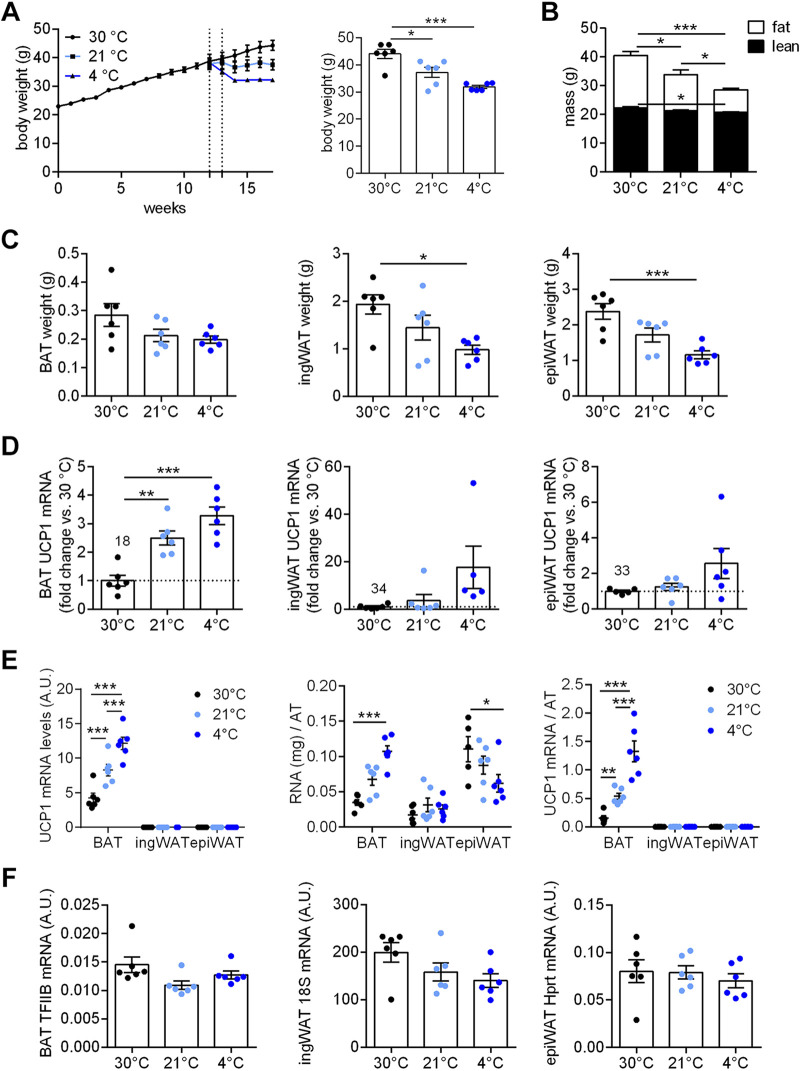
Adipose tissue recruitment in diet-induced obese mice. Mice were fed a high-fat diet at thermoneutrality for 12 wk to induce obesity. They then either remained at thermoneutrality or were exposed to semi-cold or cold conditions for 4 wk while still eating the high-fat diet. *A* (*left*): body weight time course. All mice were kept at 30°C for the first 12 wk on the high-fat diet; the first dashed line indicates the start of 18°C acclimation week for the 4°C group, second dashed line indicates the start of 4°C and 21°C periods, (*right*): final body weights. *B*: final fat and lean masses. *C*: wet weights of brown adipose tissue (BAT), inguinal white adipose tissue (ingWAT), and epididymal white adipose tissue (epiWAT). *D*: UCP1 gene expression in BAT, ingWAT, and epiWAT (expressed as fold change over the 30°C group) of the high-fat diet-fed mice after 4 wk at 30°C, 21°C, and 4°C. The values given above the 30°C bars indicate the mean *Ct* value for that condition in each tissue. The level of UCP1 mRNA in the cold in ingWAT is significantly higher (*P* ≤ 0.05) than at 30°C when analyzed with the non-parametrical Kruskal–Wallis and Dunn’s multiple comparisons test. *E* (*left*): UCP1 mRNA levels in BAT, ingWAT, and epiWAT; values presented were calculated as 2^−^*^Ct^* × 1,000,000; (*middle*): total amounts of RNA per BAT, ingWAT, and epiWAT (one outlier in epiWAT has been removed); (*right*): total amounts of UCP1 mRNA in BAT, ingWAT and epiWAT; data were obtained by multiplying the data in the *left* with the data in the *middle*. *F*: Reference gene expression levels in BAT (TFIIB), ingWAT (18S), and epiWAT (Hprt) related to data in Figs. 6*D* and 7, *A*–*C*; values are antilog-transformed *Ct* values (2^−^*^Ct^* × 1,000,000). Statistics for all panels: *n* = 5–6 mice/group, means ± SE, one-way ANOVA and Tukey’s (Sidak’s for *E*) multiple comparisons test, *P < 0.05, **P < 0.01, ***P < 0.001.

As expected, the mice exposed to the high-fat diet and maintained at thermoneutrality gained significantly in body weight: they weighed ∼45 g (Fig. 6*A*) versus ∼25 g for the nonobese (Fig. 1*A*). The increase in body weight was largely due to fat accumulation: some 20 g (Fig. 6*B*) versus less than 3 g in the nonobese (Fig. 1*A*). The lean mass was ∼20 g and largely unaffected by high-fat diet or cold acclimation (Figs. 1*A* and 6*B*).

Body weight and fat mass were markedly decreased by chronic semi-cold and cold treatment compared with the thermoneutral group (Fig. 6, *A* and *B*). ingWAT and epiWAT weights were accordingly decreased by cold treatment, whereas BAT wet weight was not significantly changed (Fig. 6*C*) (although its composition is known to be markedly altered by cold, see also e.g., Fig. 1*D*).

#### Cold-acclimation induced recruitment of UCP1 gene expression in diet-induced obese mice.

Similarly to the case in lean mice, the UCP1 mRNA levels in BAT in the diet-induced obese mice were increased due to cold acclimation, although somewhat more modestly when expressed relatively (Fig. 6*D*). This was explainable by a somewhat higher gene expression level in thermoneutral obese mice compared with nonobese (“lean”) mice, in principal agreement with earlier observations of increases in BAT recruitment due to diet-induced obesity (36–38). In the obese mice, cold acclimation did not significantly induce UCP1 gene expression in ingWAT or in epiWAT (Fig. 6*D*). The reference gene expression levels are shown in Fig. 6*F* and were not affected by housing temperature.

As seen from the *Ct* values indicated in Fig. 6*D*, the UCP1 mRNA levels (principally per mg RNA) were again much higher in BAT compared with ingWAT (∼50,000-fold at 30°C and ∼10,000-fold at 4°C) and epiWAT (∼60,000-fold at 30°C and 100,000-fold at 4°C) (Fig. 6*E*, *left*). Thus, ingWAT browning in these obese mice was impaired, principally in agreement with earlier observations (1, 9, 39).

The total amount of RNA also increased in BAT of obese mice, but not in ingWAT, and decreased in epiWAT upon cold exposure (Fig. 6*E*, *middle*). When expressed as the total amount of UCP1 mRNA per depot (Fig. 6*E*, *right*), the levels in BAT in the semi-cold- and cold-acclimated mice were approximately the same as in the lean mice (Fig. 6*E* vs. Fig. 1*D*). Thus, the thermogenic capacity estimated in this way was similar in the obese and lean mice, principally in agreement with the concept that obesity does not insulate against heat loss (40). In the cold-acclimated obese C57BL/6J mice, the estimated total thermogenic capacity of BAT (i.e., the total amount of UCP1 mRNA, Fig. 6*E*, *right*) was 3,000-fold higher than that in ingWAT.

#### Effect of cold acclimation on pro-and anti-inflammatory markers in diet-induced obese mice.

**Figure 7.**
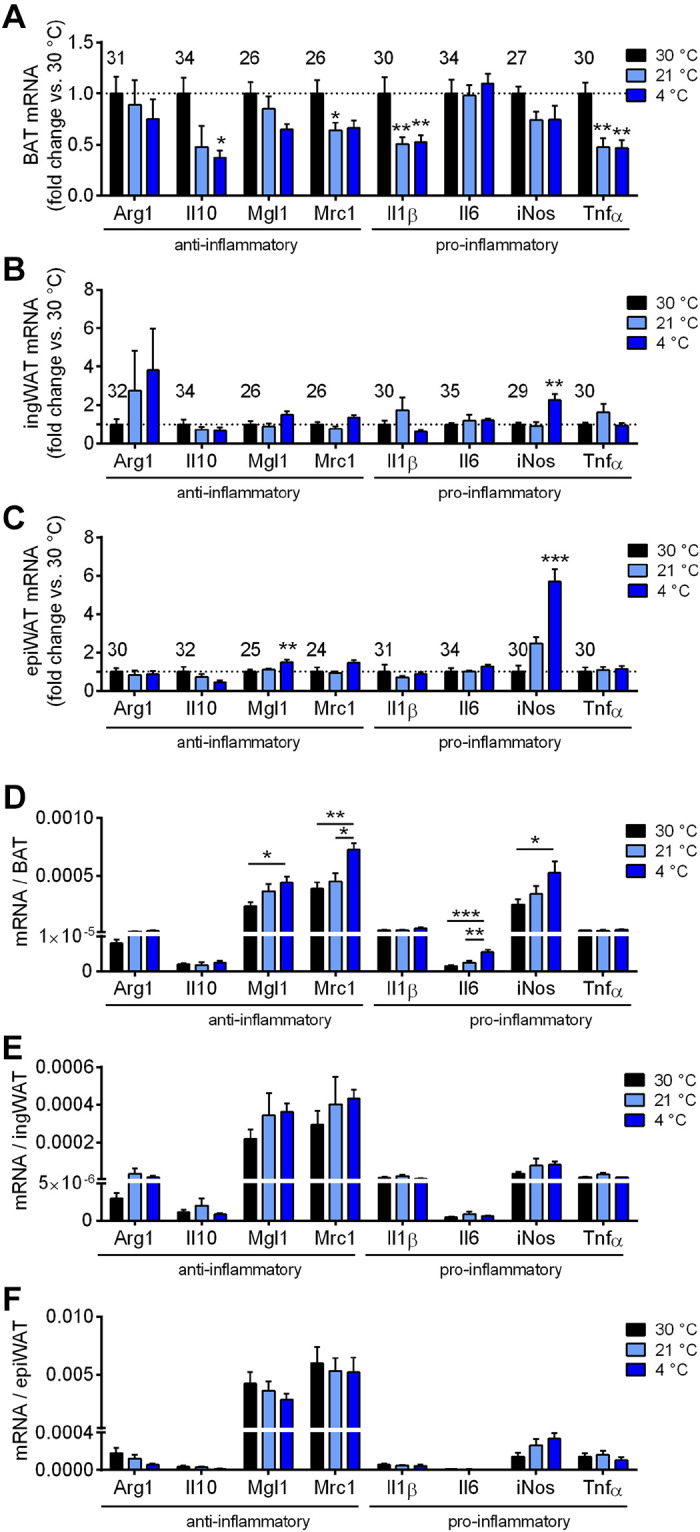
Thermogenic recruitment in obese mice is not accompanied by repression of inflammatory gene expression. The mice characterized in Fig. 6 were analyzed for pro- and anti-inflammatory markers. Gene expression of anti-inflammatory markers (Arg1, Il10, Mgl1, Mrc1) and proinflammatory markers (Il1β, Il6, iNos, Tnfα): brown adipose tissue (BAT) (*A*), inguinal white adipose tissue (ingWAT) (*B*), and epididymal white adipose tissue (epiWAT) (*C*). The values were calculated versus the reference genes shown in Fig. 6*F*. Results are expressed as fold change over the 30°C group. The value above each gene is the mean *Ct* value for the gene at 30°C. Total levels of mRNA for anti- and proinflammatory markers in BAT (*D*), ingWAT (*E*), and epiWAT (*F*) (one outlier was removed). The data were obtained in a similar way to that for UCP1 in Fig. 6*E* (*right*), i.e. by multiplying the levels of each mRNA (2^−^*^Ct^* × 1,000,000) by the total amount of RNA in the tissue (Fig. 6*E*, *middle*). Statistics for all panels: *n* = 4–6/group, means ± SE, one-way ANOVA and Sidak’s multiple comparisons test, *P < 0.05, **P < 0.01, ***P < 0.001.

To elucidate the association between the thermogenic function of the tissue and macrophage presence in these obese mice, we again determined the expression of several anti- (Arg1, Il10, Mgl1, and Mrc1) and proinflammatory (Il1β, Il6, iNos, and Tnfα) markers in the whole tissues (Fig. 7, *A–C*). The results were normalized to the thermoneutral (30°C) group. In general, the expression levels of most of these genes were still low, with many *Ct* values >30. In BAT, most of the studied markers, both anti- (Il10 and Mrc1) and proinflammatory (Il1β and Tnfα), again appeared to decrease (statistically or showing a tendency) upon semi-cold or cold exposure, compared with the 30°C group (Fig. 7*A*). However, in ingWAT and epiWAT, none of the studied markers were decreased upon cold exposure. Expression of iNos increased in ingWAT and epiWAT (Fig. 7, *B* and *C*). Thus, based again on relative levels of gene expression, the results would indicate that BAT recruitment was accompanied by a marked and consistent reduction in macrophage infiltration.

However, as seen in Fig. 6*E* (*middle*), cold acclimation resulted in a very substantial increase in total RNA in BAT [with no change in ingWAT and a decrease in epiWAT, principally in accordance with the decrease in total wet weight of epiWAT (Fig. 6*C*)]. Thus, the total expression in the tissues of these genes was calculated, as seen in Fig. 7, *D–F*. The apparent decreases in gene expression in BAT (Fig. 7*A*) were again only apparent (Fig. 7*D*), again being due to dilution by the extra RNA synthesized during tissue recruitment (Fig. 6*E*), probably mainly in the recruited brown adipocytes. Thus, with an integrative approach, we arrived at a conclusion opposite from that in a conventional analysis: BAT recruitment in obese mice was not associated with macrophage attrition but rather, if anything, with a very modest macrophage accretion.

Macrophage-associated gene expression in ingWAT and epiWAT was not influenced by cold acclimation (Fig. 7, *E* and *F*).

#### Effect of cold acclimation on macrophage number in diet-induced obese mice.

**Figure 8.**
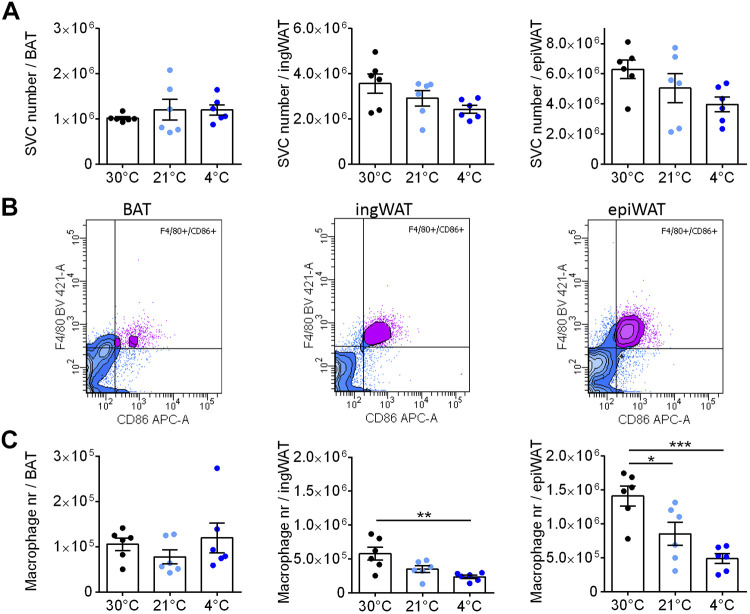
Chronic cold treatment lowers macrophage number in inguinal white adipose tissue (ingWAT) and epididymal white adipose tissue (epiWAT) in obese mice. The stromal-vascular fraction (SVC) was isolated from the adipose tissue depots characterized in Fig. 6. *A*: recovered number of SVCs per brown adipose tissue (BAT), ingWAT, and epiWAT depot. *B*: representative flow cytometry contour plots of F4/80 and CD86 from one mouse acclimated to 30°C. Macrophages were defined as F4/80+/CD86+ cells, as detailed in Fig. 3. *C*: the effect of cold acclimation on the total number of macrophages recovered from the three tissues. Statistics for (*A*) and (*C*): *n* = 6 mice/group, means ± SE, one-way ANOVA and Tukey’s multiple comparisons test, *P < 0.05, **P < 0.01, ***P < 0.001.

To examine whether there was a correspondence between pro- and anti-inflammatory gene expression and the number and type of macrophages in the obese mice, flow cytometric analyses were performed on stromal-vascular cells (SVCs) isolated from BAT, ingWAT, and epiWAT. As before, the total number of recovered SVCs in the three depots were isolated and counted. In BAT from obese mice, the total number of SVCs was about the same (≈1,000,000) as in the lean mice, but in ingWAT and epiWAT the number of cells was clearly higher than in the lean (Fig. 8*A*). In each depot, the number of SVCs was, however, not statistically significantly affected by cold acclimation (Fig. 8*A*).

As shown in Fig. 3*B*, macrophages were identified as F4/80+/CD86+ cells. An example of the flow cytometric analysis is shown in Fig. 8*B*, with data from all three depots from one mouse acclimated to 30°C. In Fig. 8*C*, the mean total number of macrophages recovered from the three tissues from the different temperature acclimation conditions is shown. As seen, at thermoneutrality, the total number is much lower in BAT (≈100,000) than in ingWAT (≈500,000) and epiWAT (≈1,500,000). Although the total number of macrophages in BAT was unchanged by cold acclimation, the number decreased in ingWAT and epiWAT (Fig. 8*C*).

#### Effect of recruitment on pro- or anti-inflammatory macrophage number in obese mice.

**Figure 9.**
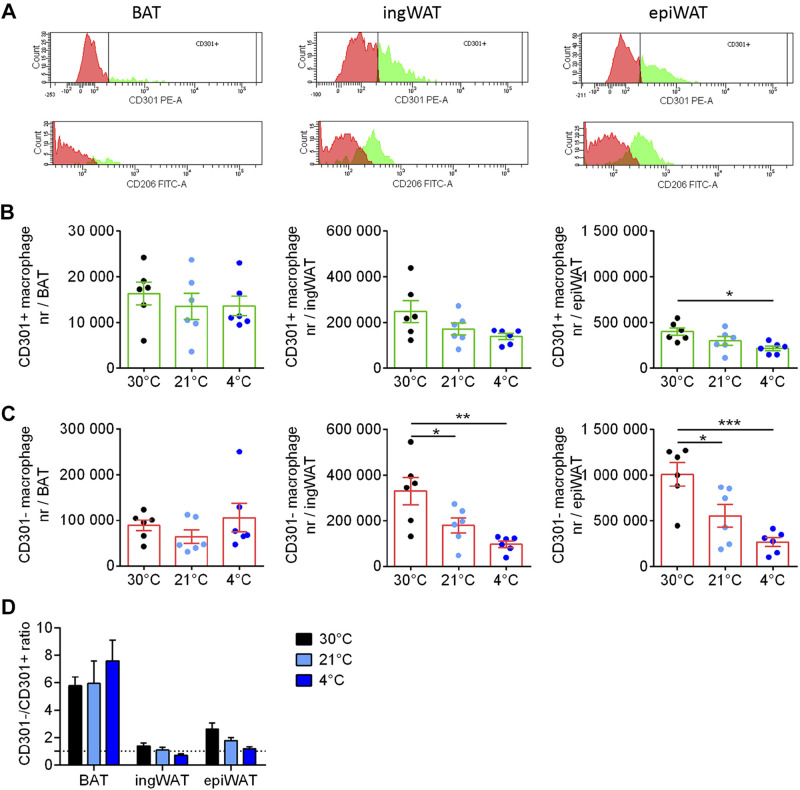
Cold acclimation lowers anti- and proinflammatory macrophage number in white adipose tissue (WAT) of obese mice. *A*: the cells that in Fig. 8*C* were identified as macrophages based on their expression of F4/80 and CD86 (F4/80+/CD86+ cells) were further analyzed here for presentation of markers identifying anti-inflammatory or proinflammatory macrophages. The analysis was performed as in Fig. 4. It is also seen here that the cells identified as anti-inflammatory based on the CD301 levels (CD301+ cells, green cells) largely distribute similarly (to the *right*) when analyzed for CD206, and those identified as proinflammatory based on the CD301 levels (orange cells) distribute to the *left* for CD206. Cells are therefore largely either CD301+/CD206+ and considered anti-inflammatory or CD301−/CD206− and considered proinflammatory. *B*: number of F4/80+/CD86+/CD301+ (anti-inflammatory macrophages) and (*C*) number of F4/80+/CD86+/CD301− (proinflammatory macrophages) per brown adipose tissue (BAT, *left*), inguinal WAT (ingWAT, *middle*), and epididymal white adipose tissue (epiWAT, *right*). *D*: Ratio of CD301−/CD301+ macrophages (pro-/anti-inflammatory) ratio in BAT, ingWAT, and epiWAT. Statistics for all panels: *n* = 6 mice/group; means ± SE, one-way ANOVA and Tukey’s (Sidak’s for *D*) multiple comparisons test, *P < 0.05, **P < 0.01, ***P < 0.001.

Similarly to the lean mice, CD301 and CD206 markers were used to identify anti- and proinflammatory macrophages. As shown in Fig. 9*A*, F4/80+/CD86+/CD301+ macrophages were again largely also CD206+; thus, only the CD301+ anti- and CD301− proinflammatory macrophage quantification is discussed below.

In BAT, the number of CD301+ (anti-inflammatory) macrophages was unchanged by recruitment but tended to decrease in ingWAT and epiWAT (Fig. 9*B*). In BAT, the number of CD301− (proinflammatory) macrophages was also unchanged by recruitment but it was clearly decreased in ingWAT and epiWAT (Fig. 9*C*). Thus, the ratio of pro-/anti-inflammatory macrophages was not markedly affected by cold acclimation in any of the three adipose tissue depots (Fig. 9*D*).

### The Relationship between Macrophage Number and Tissue Size

**Figure 10.**
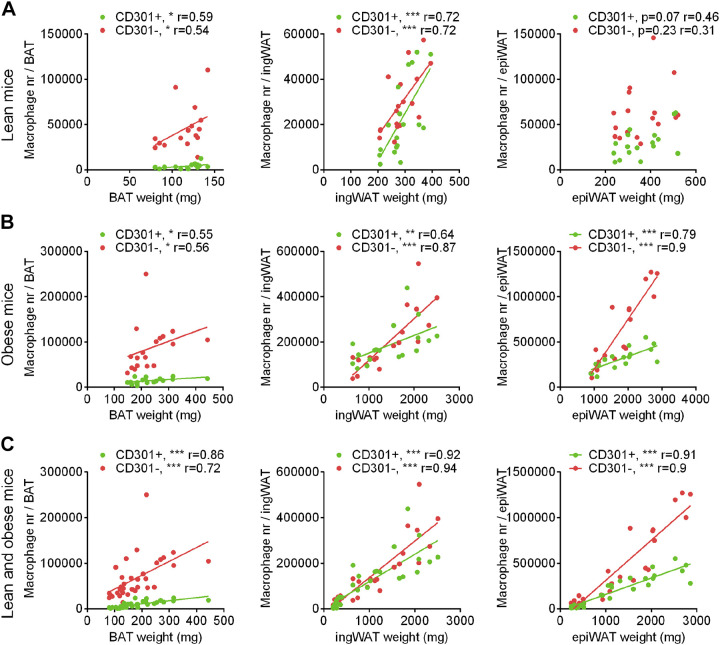
Correlations between adipose tissue depot weight and macrophage number. For each mouse examined in the previous figures, the numbers of pro- or anti-inflammatory macrophages were plotted against the adipose tissue weight of that mouse, irrespective of acclimation temperature in non-obese mice from Figs. 1–4 (*A*) and diet-induced obese mice from Figs. 6-9 (*B*). Statistics: *n* = 17–18 mice, Spearman correlation test, *P < 0.05, **P < 0.01, ***P < 0.001. *C*: The data from (*A*) and (*B*) combined.

If macrophage accretion or attrition is involved in regulation of BAT and WAT function, including the acquisition of thermogenic capacity, it may be posited that the number should be a function of recruitment state rather than simply reflecting tissue size. To examine whether tissue size is associated with total macrophage number in the tissue, we have plotted in Fig. 10, *A* and *B* tissue weight versus macrophage number for all conditions examined here (*A* for nonobese and *B* for obese mice). It is evident from these plots that there was in general a simple and highly significant correlation between tissue size and both pro- and anti-inflammatory macrophage number; one exception (i.e., nonsignificant) was the epiWAT depot in the lean mice but even here the tendency was the same. Although these correlations do not exclude a determinative role for macrophages in tissue recruitment (through their activity), the data would be compatible with a tenet that macrophages are aides to tissue function that are dispersed throughout the tissue volume. It may be noted that the pro- and anti-inflammatory macrophages did not display opposing patterns of dependence on tissue weight, despite their expected opposing functions. It can still clearly be proposed that the high number of proinflammatory macrophages seen, e.g., in the epiWAT depot in the obese mice would, at a systemic level, convey detrimental effects to health.

Although there was an age difference between the obese and nonobese mice, we have still allowed ourselves in Fig. 10*C* to plot all the data together for each depot. As seen, over a wide range of tissue weights for each depot—and thus irrespective of recruitment state or obesity as such—the number of both anti- and proinflammatory macrophages remain a function of tissue size. There was apparently no reinforcing effect of increasing obesity on macrophage number in the epiWAT depot. Thus, with obesity, we find no indication that epiWAT per se attains a more inflammatory state.

### No Relationship between Macrophage Recruitment and Browning of Adipose Tissues

**Figure 11.**
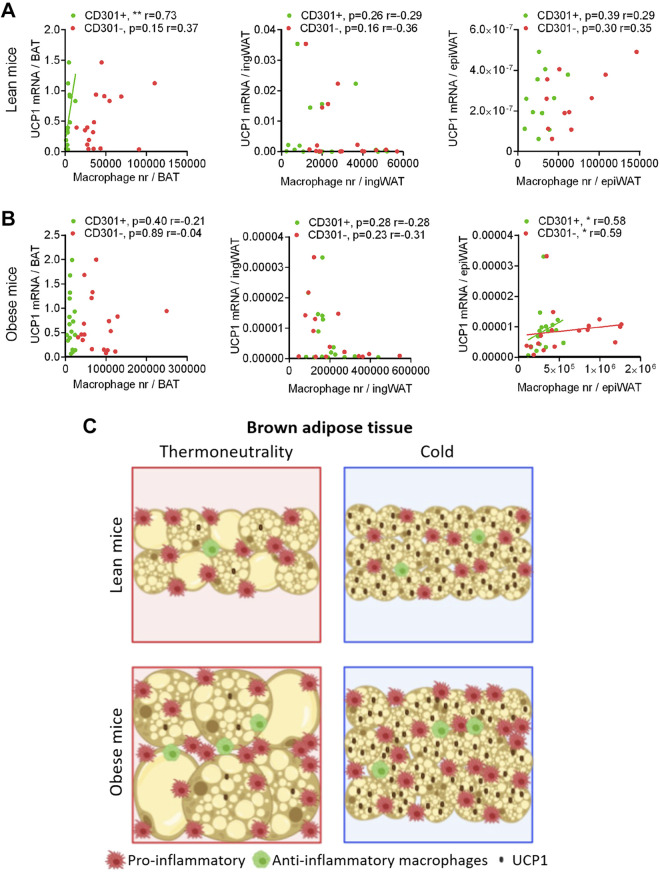
Lack of correlation between macrophage number and the browning of adipose tissues. The data for total UCP1 mRNA levels in the adipose tissues displayed in the earlier figures are plotted here against the corresponding total number of pro- or anti-inflammatory macrophages in that tissue, separately for the nonobese mice (*A*) (in the inguinal white adipose tissue (ingWAT) and epididymal white adipose tissue (epiWAT) plots, two outliers were removed) and the diet-induced obese mice (*B*) (in the ingWAT and epiWAT plots, data from one outlier mouse were removed). Statistics: *n* = 17–18 mice (lean epiWAT *n* = 11), Spearman correlation test, *P < 0.05, **P < 0.01. *C*: summary of events in brown adipose tissue (BAT): in cold-induced recruitment, the total amount of UCP1 increases extensively but the number of proinflammatory macrophages does not change; the number of anti-inflammatory macrophages is somewhat increased but only in the lean mice. The area of the tissue drawn illustrates the change in total tissue amount.

The major motivation behind the present investigation was to examine to which degree pro- or anti-inflammatory macrophages in BAT and ingWAT could be ascribed a regulatory and mediatory role for the recruitment process and its maintenance. In general, the mechanisms suggested earlier would either indicate that recruitment was associated with a reduced number of proinflammatory macrophages or with a higher number of anti-inflammatory macrophages. To examine such relationships, in Fig. 11 we have collected all data concerning total UCP1 mRNA levels in the different tissues and analyzed to what degree these levels correlated with pro- or anti-inflammatory macrophage number in the different tissues and conditions.

As seen, in the ingWAT, there was no correlation at all between thermogenic recruitment and macrophage number (Fig. 11, *A* and *B*, *middle*). In obese epiWAT, there was a statistically significant positive correlation, but total UCP1 mRNA amounts in this depot are so low that it is not physiologically meaningful (Fig. 11*B*, *right*). However, in BAT of lean mice (Fig. 11*A*, *left*), there was a clear positive correlation between UCP1 mRNA amount and the number of anti-inflammatory macrophages. Taken alone, this could be considered evidence for a recruitment-promoting effect of anti-inflammatory macrophages. However, as seen in Fig. 11*B* (*left*), this correlation was not observed in obese mice. We also found (Fig. 5) that the increase in anti-inflammatory macrophages in BAT was dependent on the presence of β_1_/β_2_-adrenergic receptors—but that tissue recruitment occurred to the same extent in the absence of this increase in macrophage number.

Thus, we have been unable to demonstrate an involvement of macrophages in maintenance of the recruited state of brown or brite/beige adipose tissues. Due to the dynamic equilibrium nature of the recruited state, this would imply that changes in macrophage number or subtype are probably not obligatory for the acquirement of this state either.

## DISCUSSION

Maintenance of a thermogenically recruited state in brown and brite/beige adipose tissues is a dynamic equilibrium process where a constant stimulation (classically induced by cold) is required to sustain the state; in the absence of stimulation, the tissues revert to the thermogenically near-dormant state observed at thermoneutrality. Here, we have especially examined the possible involvement of macrophages in maintenance of the recruited state of thermogenic adipose tissues, a role that would be mediated either via a reduced number of proinflammatory macrophages and/or by a higher number of anti-inflammatory macrophages. To simplify the discussion, we use the terms anti- and proinflammatory, well aware that these are oversimplifications.

### Thermogenic Recruitment and Macrophage Roles in BAT

Direct enumeration of proinflammatory macrophages in BAT demonstrated no consistent effect of recruitment on this parameter (Fig. 8*C*). In apparent contrast, relative gene expression indicated that the expression of proinflammatory macrophage-associated genes was decreased in BAT in the recruited state. However, calculation of gene expression per whole fat depot concurred with the outcome of direct enumeration in that no alterations in gene expression were seen.

Concerning the possibility that accretion of anti*-*inflammatory macrophages may be an intermediate in the recruitment process, we observed a clear increase in the number of anti-inflammatory macrophages in recruited BAT, although only in the nonobese mice. Since thermogenic recruitment proceeded unhindered also in the obese mice, thermogenic recruitment cannot be secondary to accretion of anti-inflammatory macrophages. This conclusion is confirmed by the observation that although accretion of anti-inflammatory macrophages was abolished in the BAT of mice lacking β_1_/β_2_-adrenoceptors, the thermogenic recruitment process was unperturbed in these mice. Our observations thus concur with earlier studies reporting an accretion of anti-inflammatory macrophages in certain recruitment conditions (20) but diverge from those in not ascribing this accretion an obligatory regulatory role for thermogenic recruitment of BAT.

Taken together, we find no indications that macrophage accretion or attrition is an obligatory and integral part of maintaining the thermogenic recruited state in BAT. As this is a dynamic state, this also implies that macrophage accretion or attrition is not mediatory for the recruitment process.

### Thermogenic Recruitment and Macrophage Roles in Brite/Beige Adipose Tissue

Much interest concerning macrophage involvement in thermogenic recruitment has centered on brite/beige adipose tissue. We have therefore also investigated the involvement of macrophages in the maintenance of the recruited thermogenic state in ingWAT. In lean mice, cold exposure clearly increased UCP1 mRNA levels in ingWAT, but this thermogenic recruitment was not associated with significant alterations in the numbers of either pro- or anti-inflammatory macrophages. In obese mice, cold acclimation was associated with a decreased number of proinflammatory macrophages in ingWAT, but this was not accompanied by thermogenic recruitment; rather this probably reflected a decreased amount of inguinal adipose tissue. Thus, we saw no evidence that browning of ingWAT was mediated by a reduction in proinflammatory macrophages or the associated cytokines (e.g., TNFα), as has been suggested (41, 42). Similarly, we saw no compelling evidence for the involvement of anti-inflammatory macrophages in the browning process in ingWAT where these macrophages have been suggested to play a mediatory role (14–17, 20, 21, 28, 29, 43).

It may be added that thermogenically, alterations in the degree of browning in brite/beige adipose tissues would be of minor importance, as the contribution of brite/beige adipose tissues to systemic thermogenesis even in the cold is very much lower than that of BAT, at least when estimated from UCP1 protein (44) or mRNA amounts (as also observed here).

### Macrophage Roles in Non-Browning WAT

As expected, we saw no thermogenic recruitment due to cold acclimation in the epiWAT of either nonobese or obese mice, nor did we see any effect of cold acclimation on pro- or anti-inflammatory macrophage number in nonobese mice. We did observe a reduction in macrophage number due to cold acclimation in obese mice (both pro- and anti-inflammatory), but cold acclimation decreased the epididymal adipose tissue depot size and the reduction in macrophage number paralleled that decrease.

### Possible Problems in Determining Macrophage Number

In adipose tissues, an accumulation of macrophages around dying adipocytes has frequently been observed and named “crown-like structures” (45). It may be discussed whether the macrophages associated with these structures would be recovered by the method used here for tissue dissociation, that is based on enumeration of macrophages in the stromal-vascular fraction. It could be envisaged that the macrophages, together with the adipocyte remnant that they degrade, would remain in the upper fat layer, if the collagenase treatment were unable to release the macrophages from the lipid. The macrophages in the crown-like structures appear even to form a syncytium. However, whereas this issue—if correct—could markedly affect the number of macrophages counted, it would not affect the expression level of macrophage-associated genes in the whole tissue. As seen from the data, these values generally accord with the corresponding macrophage numbers. Thus, it would not seem that the occurrence of crown-like structures would markedly influence the outcome of the present study.

### The Effect of Obesity on Macrophage Number in Adipose Tissues

We did observe a higher total number of macrophages (both pro- and anti-inflammatory) in the adipose tissues of the obese mice. Such accumulation is often considered evidence for an inflammatory state due to obesity, but we observed that the increased number of macrophages seemed to be largely explainable by the increased total mass of the adipose tissue depots. Also specifically in BAT, we saw an increased number of macrophages in obesity but found no evidence for macrophage infiltration that is not understandable on the basis of tissue enlargement (i.e., not as discussed in Ref. 8).

In terms of the types of macrophages that accumulate in the obese state, we did not see a markedly changed balance between pro- and anti-inflammatory macrophages in BAT; in thermoneutral mice, the predominance of pro- over anti-inflammatory macrophages was higher in the obese mice than in the nonobese mice—but under semi-cold (21°C) and cold (4°C) conditions, there is no marked difference. In epiWAT, we did not see the shift from a predominantly anti- to a predominantly proinflammatory macrophage population that has been implied from earlier studies (8). Similarly, when gene expressions were followed, we saw no major increase in the expression levels of proinflammatory mediators in the obese versus the lean mice.

In addition, analysis of the implied physiological significance of the results is, as with other topics, dependent upon how these results are expressed. When quantifying adipose tissue macrophage numbers in response to thermogenic recruitment, a physiological systemic “endocrine” analysis can be applied. Thus, macrophages—particularly the proinflammatory—may be considered as sources of cytokines, etc., that are released into the circulation and that may negatively affect the function of other organs (e.g., decrease muscle insulin sensitivity). Given our findings that total macrophage numbers in adipose tissues change principally in direct proportion to the growth or reduction of the tissue, thermogenic recruitment would lead to amelioration of the health status of the organism.

Macrophage number can also be expressed as a tissue density. Since we find that the BAT weight in the nonobese mice is not much changed by recruitment, we conclude that the proinflammatory macrophage density in BAT is the same across recruitment states. Although this was not observed in immunohistochemistry, that rather indicated higher macrophage densities in thermoneutral mice (46, 47), the presence of the macrophages may then represent their cognate participation in the remodeling process in the tissue (48, 49), rather than pointing to a mediatory role.

However, in the analysis of tissue density, the functional difference between the recruitment states is not considered. At thermoneutrality, much of BAT consists of triglyceride droplets (1). The recruitment process in BAT leads to a many-fold increase in the density of cells and proteins in the tissue (44, 50). Thus, if a mediatory paracrine effect is considered, the cytokines/macrophages would better be expressed per target cell. The conclusion would then be that recruitment leads to a marked decrease in macrophage number per target cell, particularly for the proinflammatory but also for the anti-inflammatory macrophages. However, this would be a consequence of the recruitment process rather than a cause.

### Macrophage Number and Gene Expression

Although we generally observed similar numbers of macrophages under the different recruitment conditions, their degree of activation may alter gene expression levels and thus e.g., secretion of cytokines, etc. We have especially followed gene expression of some macrophage-associated genes that have earlier been discussed in relation to BAT and brite/beige adipose tissue recruitment: TNFα, IL6, iNOS, and IL1β. In our present analysis, we cannot ascertain whether these genes are expressed in the macrophages or e.g., in the parenchymal cells of the tissues.

Although it has been reported that thermogenic recruitment is associated with or even partially mediated by a decrease in TNFα, our integrative analysis of TNFα gene expression in BAT found no effect of recruitment on TNFα mRNA levels, in contrast to earlier suggestions (9, 18, 21, 51–53). Thus, our results did not support the suggestion that recruitment is (partially) mediated through alleviation of inhibition caused by TNFα.

The cytokine interleukin-6 (IL6) is traditionally seen as proinflammatory, although this classification may be discussed (54). We found it to be expressed at very low levels but the levels were somewhat increased in the recruited state in both BAT and ingWAT. It has been indicated that its expression in the tissue under certain conditions may be systemically important (55), although the very-low expression levels make a mediatory role in the recruitment process less likely.

The enzyme iNOS (inducible NO synthase) has also been considered to have a negative effect on BAT recruitment (56) but we found very modest changes in mRNA levels, and in a direction contrary to what would be anticipated, and it is therefore unlikely that it is directly involved in the recruitment process.

IL1β (interleukin 1β) has also been reported to inhibit UCP1 gene expression (57, 58) but the gene expression levels were very stable in the different recruitment states examined here and it therefore also seems unlikely that it is involved in the recruitment process.

In a general analysis of each of the six datasets (effects of cold acclimation in three tissues and two conditions (lean and obese)), we found 15 significant correlations (out of 48 possible) between UCP1 mRNA levels and the mRNA levels of the 8 mediators followed here (not detailed). However, we found no systematic pattern: no mediator was consistently (positively or negatively) associated with increased recruitment (UCP1 mRNA levels) in any conditions or tissues.

### A Role versus a Regulatory Role

As can be understood from the above, we have not identified such alterations in the macrophage populations of brown or brite/beige adipose tissues that would make it probable that they play a decisive role in the maintenance of the thermogenic recruitment state—or, given the dynamic nature of the recruited state, in the recruitment process as such. This does not, of course, indicate that there is no influence of the existing macrophages on BAT and brite/beige adipose tissue function. Thus, negative or positive modulators may indeed be secreted from the macrophages. Pharmacologically induced alterations in macrophage number may also affect the tissues. Similarly, treatment of brown, brite/beige and white adipocytes in vitro with cytokines or other substances that could potentially emanate from macrophages may affect these cells. The macrophages presumably also perform the tissue remodeling processes that they are generally associated with, particularly in transition states. What we can conclude is that, contrary to our expectations, we have been unable to ascertain that in the dynamic equilibrium that the thermogenically recruited state represents, there would appear to be a mediatory role of the tissue macrophages.

## GRANTS

This study was supported by a Grant from The Swedish Research Council. N. Boulet was the recipient of a postdoctoral scholarship from The Department of Molecular Biosciences, The Wenner-Gren Institute.

## DISCLOSURES

No conflicts of interest, financial or otherwise, are declared by the authors.

## AUTHOR CONTRIBUTIONS

N.B., B.C., and J.N. conceived and designed research; N.B. and I.H.N.L. performed experiments; N.B., B.C., and J.N. analyzed data; N.B., B.C., and J.N. interpreted results of experiments; N.B. prepared figures; N.B. drafted manuscript; N.B., I.H.N.L., B.C., and J.N. edited and revised manuscript; N.B., I.H.N.L., B.C., and J.N. approved final version of manuscript.
